# 

**Published:** 1887-05

**Authors:** 


					﻿/vo£ yii
MAY, 1887.
NO. 5.
JPHE
Homeopathic pn Y^iciAp,
A MONTHLY JOURNAL OF MEDICAL SCIENCE.
“ He is a freeman whom truth, makes free, and all are slaves beside."
"Seek the Truth: come whence it may, cost what it will."
EDITED BY
Edmund J. lee, m. d.,
AND
WALTER M. JAMES, ME. D.
PHILADELPHIA:
No. 1123 SPRUCE STREET.
asr e w .Yomc
A. L. CHATTERTON & COMPANY, Publisher's Agents.
LONDO KT:
ALFRED HEATH & CO., Sole Agents FOB Great Britain,
114 Eb ary Street, Eaton Square.
I early Subscription, $2.50, invariably in advance. Single numbers, 25 cts.
Entered at the Philadelphia Post-Office aa Second-Class Rail Matter.
				

